# Perfusion reduces bispecific antibody aggregation via mitigating mitochondrial dysfunction-induced glutathione oxidation and ER stress in CHO cells

**DOI:** 10.1038/s41598-020-73573-4

**Published:** 2020-10-06

**Authors:** Pritam Sinharoy, Aaron H. Aziz, Natalia I. Majewska, Sanjeev Ahuja, Michael W. Handlogten

**Affiliations:** 1 Cell Culture and Fermentation Sciences, AstraZeneca, Gaithersburg, MD USA; 2 Department of Chemical and Biomolecular Engineering, Whiting School of Engineering, Johns Hopkins University, Baltimore, MD USA

**Keywords:** Cell biology, Chemical engineering

## Abstract

One major challenge observed for the expression of therapeutic bispecific antibodies (BisAbs) is high product aggregates. Aggregates increase the risk of immune responses in patients and therefore must be removed at the expense of purification yields. BisAbs contain engineered disulfide bonds, which have been demonstrated to form product aggregates, if mispaired. However, the underlying intracellular mechanisms leading to product aggregate formation remain unknown. We demonstrate that impaired glutathione regulation underlies BisAb aggregation formation in a CHO cell process. Aggregate formation was evaluated for the same clonal CHO cell line producing a BisAb using fed-batch and perfusion processes. The perfusion process produced significantly lower BisAb aggregates compared to the fed-batch process. Perfusion bioreactors attenuated mitochondrial dysfunction and ER stress resulting in a favorable intracellular redox environment as indicated by improved reduced to oxidized glutathione ratio. Conversely, mitochondrial dysfunction-induced glutathione oxidation and ER stress disrupted the intracellular redox homeostasis, leading to product aggregation in the fed-batch process. Combined, our results demonstrate that mitochondrial dysfunction and ER stress impaired glutathione regulation leading to higher product aggregates in the fed-batch process. This is the first study to utilize perfusion bioreactors as a tool to demonstrate the intracellular mechanisms underlying product aggregation formation.

## Introduction

Bispecific antibodies (BisAbs) are a class of monoclonal antibodies (mAbs) designed to recognize two different antigens, making them capable of targeting multiple pathways simultaneously^1^. Over the past few years, BisAbs have increasingly become popular in the clinic for the treatment of numerous human health disorders. According to a recent report, more than 85 BisAbs are being considered for clinical development, with a majority of these drugs being evaluated for the treatment of cancer^1^. Unfortunately, BisAb development and production is often challenged by manufacturing problems due to low production yields^2^. Specifically, a common challenge observed during the production of BisAbs is high product aggregates (exceeding 40% in some cases) due to mis-pairing of engineered disulfide bonds^3–5^. Product aggregates can trigger immunogenic responses in patients and therefore must be removed, severely decreasing product yields and increasing commercial manufacturing costs. Although product aggregates are formed intracellularly, traditional process development strategies to mitigate product aggregation focus on extracellular parameters (pH, temperature, DO etc.), virtually ignoring their effects on the intracellular environment^6,7^. Additionally, a recent study demonstrated that a perfusion process decreased product aggregate levels compared to a fed-batch process^8^. However, at present, the fundamental mechanisms leading to intracellular BisAb aggregate formation remain unknown.

BisAbs contain engineered disulfide bonds for structural stability, which have been previously implicated in BisAb aggregation^3^. It is well known that intermolecular/intramolecular disulfide bond formation is sensitive to the intracellular redox state. The intracellular redox environment is primarily influenced by the intracellular free thiol content (cysteine and glutathione) and reactive oxygen species (ROS). ROS are constantly generated intracellularly by partial reduction of oxygen primarily during mitochondrial oxidative phosphorylation and oxidative protein-folding in the endoplasmic reticulum (ER). Oxidative stress, due to overwhelming ROS production and accumulation, can damage cellular structure and functions which ultimately affect the intracellular redox-homeostasis. Hence, the fundamental understanding of the coupling between the intracellular redox environment and disulfide bond formation in the ER is essential to mitigate product aggregate formation during BisAb production.

In this study we utilized fed-batch and perfusion processes as tools to determine the underlying intracellular mechanisms that influence BisAb aggregate formation in the ER. Our hypothesis was that oxidative stress-induced aberrant redox homeostasis contributes to higher aggregate formation in a fed-batch process than a perfusion process. This is the first study to investigate and compare fed-batch and perfusion bioreactors at the intracellular levels to gain a perspective on the key regulators contributing to intracellular aggregate formation. Therefore, we cultured the same clonal CHO cell line producing a BisAb in both fed-batch and perfusion processes for 16–20 days. We compared the intracellular redox environment of both processes by investigating mitochondrial contribution to ROS, intracellular glutathione levels and protein folding in the ER. Briefly, our data demonstrate that higher specific productivity in fed-batch bioreactors caused mitochondrial dysfunction and ER stress. Mitochondrial dysfunction exaggerated intracellular ROS production causing excessive glutathione oxidation in fed-batch bioreactors. Imbalanced intracellular glutathione regulation coupled with ER stress led to mis-paired disulfide bond formation, leading to increased intracellular BisAb aggregate formation in fed-batch bioreactors. Comparatively, the perfusion process mitigated mitochondrial dysfunction and ER stress. Mitigation of mitochondrial dysfunction improved intracellular glutathione homeostasis and attenuated mis-paired disulfide bond formation, causing decreased intracellular BisAb aggregate formation in perfusion bioreactors.

## Results

### Perfusion process increases viable cell density (VCD) and decreases specific productivity

**Figure 1:**
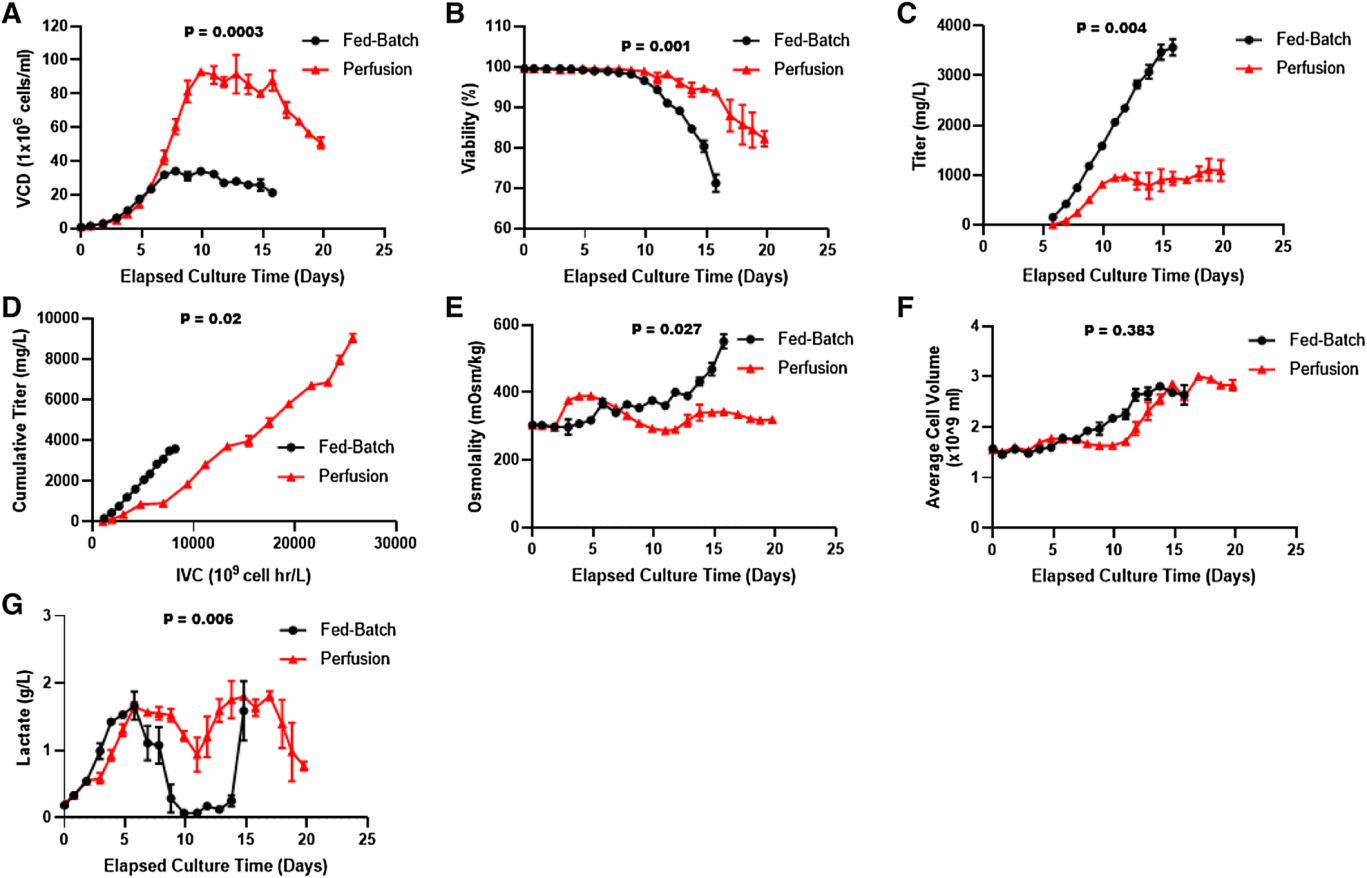
Comparison of process analysis between fed-batch and perfusion processes for BisAb production. Samples from each bioreactor were taken every day to measure (**A**) viable cell density, (**B**) cell viability, (**C**) day-day titer, (**D**) specific productivity, (**E**) osmolality, (**F**) cell volume and (**G**) lactate concentration. All data are expressed as mean ± SD from duplicate bioreactors.

The hypothesis was investigated by running fed-batch and perfusion bioreactors with the same clonal CHO cell line expressing a BisAb. The process conditions for both CHO cell culture processes are outlined in Table 1. Our data demonstrates that the perfusion process increased peak VCD by ~ threefold when compared to the fed-batch process (Fig. 1A). Perfusion bioreactors also maintained higher CHO cell viability throughout the cell culture process compared to fed-batch bioreactors (Fig. 1B). Day-to-day titer concentrations (Fig. 1C) and cell specific productivity (Fig. 1D) throughout the experiment were markedly lower in perfusion bioreactors compared to fed-batch bioreactors. However, the cumulative titers were ~ 2.5-fold higher in the perfusion process compared to the fed-batch process (9.0 ± 0.2 g/l vs. 3.6 ± 0.1 g/l). A previous study has shown the negative implications of higher culture media osmolality levels on cell growth and viability in a mAb producing CHO cell line^9^. In our study, the initial osmolality of the culture media at day 0 for both fed-batch and perfusion processes were the same (Fig. 1E). Osmolality levels began to increase after day 10 in fed-batch bioreactors, whereas, it remained constant in perfusion bioreactors. The average cell volume in fed-batch bioreactors were comparatively higher than perfusion bioreactors during the stationary phase, however, we did not observe any significant difference in cell size between the processes overall (Fig. 1F). Both fed-batch and perfusion bioreactors had favorable lactate profiles, peaking at < 2 g/l (Fig. 1G). In summary, the perfusion process increased peak VCD, maintained higher cell viability, and decreased cell specific productivity compared to the fed-batch process.

**Table 1:** Process parameters for fed-batch and perfusion processes.

Parameters	Fed-batch	Perfusion
Temperature (°C)	35.5	35.5
pH ± dead band	7.0 ± 0.1	7.0 ± 0.1
% Dissolved oxygen	50	40
Agitation (RPM)	300	300
Glucose levels	Maintain at > 0.5 g/l	Maintain at > 0.5 g/l
Feed timing	Bolus feed every other day starting from day 3	NA
Perfusion rate	NA	1 VVD starting from day 2

The same clonal CHO cell line expressing a BisAb was cultured using either a fed-batch or a perfusion process.

The processes were run in duplicate 3 l bioreactors. Cells were inoculated at 0.8 × 10^6^ cells/ml.

### Perfusion process mitigates intracellular BisAb aggregate formation

**Figure 2:**
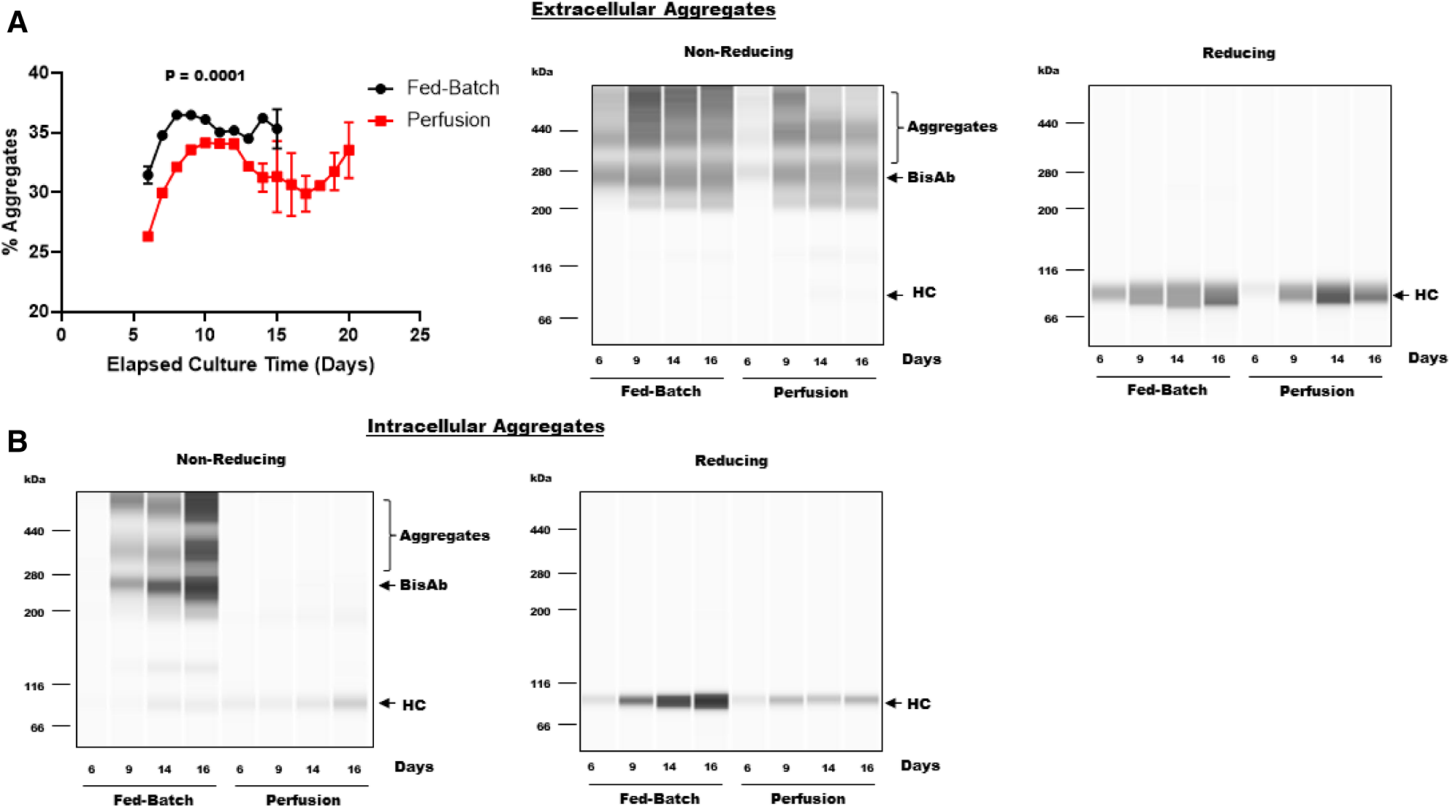
Analysis of BisAb aggregate formation in fed-batch and perfusion processes. (**A**) Left Panel: Cell free samples were collected from each bioreactor from day 6 onwards. Samples were analyzed by ultra-performance size exclusion chromatography to determine the percentage of total BisAb aggregates. Aggregate data are expressed as mean % of the total product ± SD from duplicate bioreactors. The cells continue to secrete both monomer and aggregates over the length of the cell culture process. Right Panel: Cell free samples collected from a fed-batch and a perfusion bioreactor on day 6, 9, 14 and 16 were analyzed by capillary western blot analysis. Media aggregate accumulation, BisAb and HC expression were determined under non-reducing (left panel) and reducing (right panel) conditions. (**B**) Cell pellets were collected from fed-batch and perfusion bioreactors on days 6, 9, 14, and 16. Intracellular aggregate accumulation, bisAb and HC expression were analyzed by capillary western blot analysis under non-reducing (left panel) and reducing (right panel) conditions.

Previously, perfusion has been shown to decrease product aggregates levels^8^. Thus, both extracellular (culture media supernatant) and intracellular (cell pellets) product aggregates were analyzed starting on day 6 of production. Throughout the culture process, aggregate levels were significantly higher in the fed-batch process compared to the perfusion process (Fig. 2A, left panel). In fed-batch bioreactors, extracellular aggregates for days 6 and 15 were 31.4 ± 0.7% and 35.3 ± 1.6% respectively (Fig. 2A, left panel). In comparison, extracellular aggregates in perfusion bioreactors on days 6 and 15 were 26.3 ± 0.3% and 31.2 ± 1.1% respectively. Extracellular aggregate levels in both cell culture processes increased during the exponential growth phase of the culture. However, the aggregate levels remained elevated in the fed-batch process during the stationary phase while aggregate levels decreased in the perfusion process. We also determined the effect of the culture medium on extracellular BisAb aggregate formation (Supplementary Fig. S1). Our data demonstrate that the culture medium had no significant effect on extracellular BisAb aggregate formation in both fed-batch and perfusion processes for 2 and 4 days after collection. Additionally, we also determined relative changes in extracellular aggregate levels using capillary western blot analysis (Fig. 2A, right panel). Under the non-reducing condition we found that aggregate levels were relatively higher in the fed-batch process compared to the perfusion process between day 6 and day 16. The reducing condition demonstrated that mispairing of disulfide bonds lead to bisAb aggregate formation as only free HC are detected post the reduction of disulfide bonds. Next, we analyzed the relative changes in intracellular accumulation of the BisAb IgG by western blot analysis under non-reducing and reducing conditions to determine the effects of perfusion on intracellular aggregate accumulation (Fig. 2B). The non-reducing western blot demonstrated that intracellular aggregate accumulation increased dramatically from day 6 to day 16 in fed-batch bioreactors whereas, only a minimal increase was observed in intracellular aggregate accumulation in perfusion bioreactors. The reducing western blot illustrated that BisAb aggregates are formed intracellularly due to mis-paired disulfide bonds, as only free HC is detected under reducing western blot (Fig. 2B). Additionally, the HC was well expressed in both processes, although at a comparatively lower level in perfusion bioreactors than fed-batch bioreactors. Together, these findings demonstrate that BisAb aggregates are formed due to mis-paired disulfide bonds, are formed intracellularly and are secreted. This observation is in accordance with studies reported previously^10^. Moreover, compared to the fed-batch process, the perfusion process demonstrated lower intracellular aggregate formation resulting in lower product aggregates in the supernatant.

### Perfusion process protects CHO cells against ROS-induced oxidative damage

**Figure 3:**
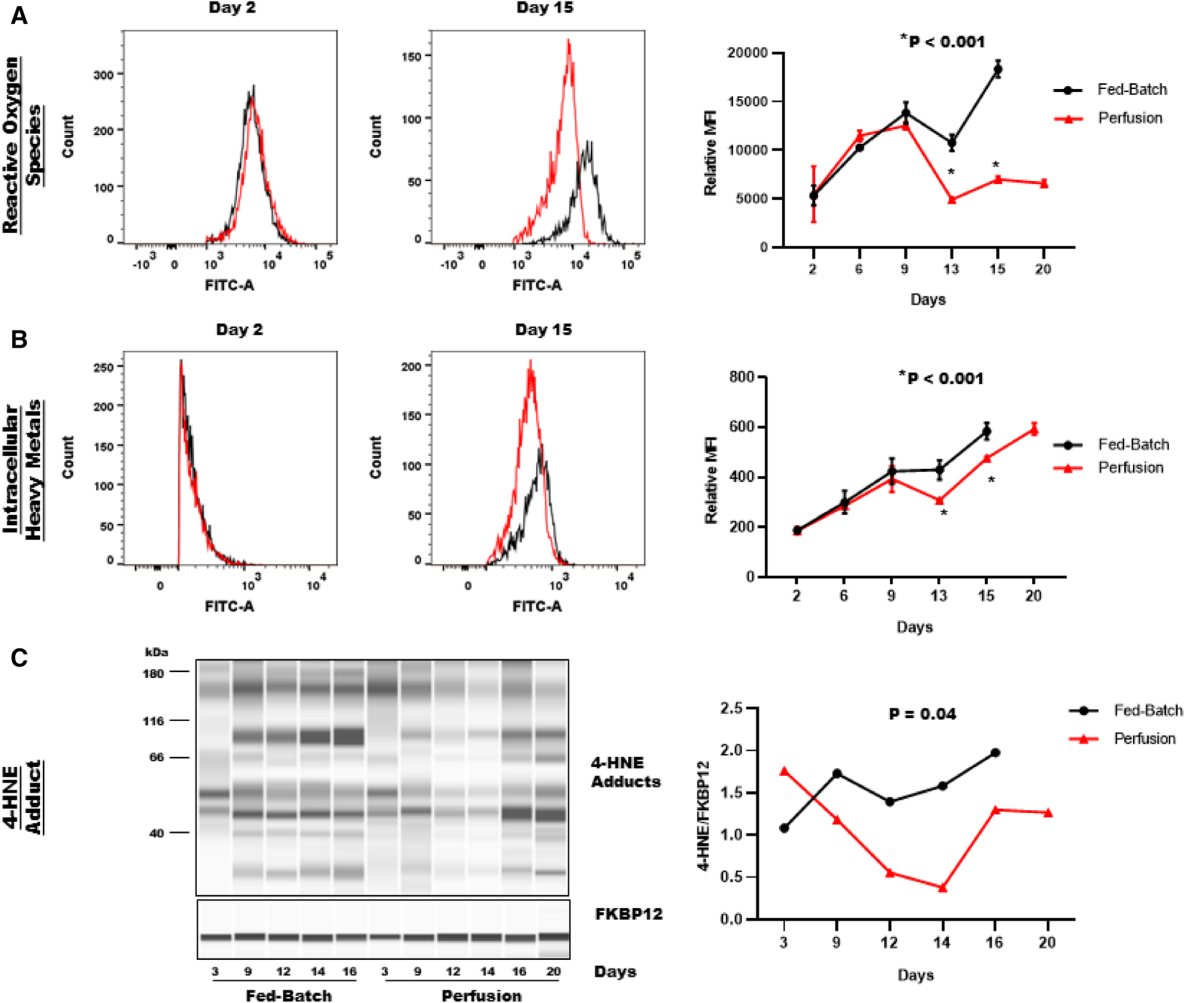
Comparison of intracellular oxidative-stress between fed-batch and perfusion bioreactors. Cell samples were taken on the indicated days to determine intracellular changes in (**A**) reactive oxygen species (ROS), (**B**) heavy metal ions and (**C**) 4-HNE adducts formation. (**A**,**B**) Representative histograms for a fed-batch and a perfusion bioreactor on day 2 and day 15 are shown in the left panel. Mean fluorescence intensity (MFI) from duplicate bioreactors are shown in the right panel. Data are expressed as mean ± SD. (**C**) Representative capillary western blot image for 4-HNE adduct formation in a fed-batch and a perfusion bioreactor is shown in the left panel. FKBP12 (~ 12 kDa) was used as loading control. Data is expressed as 4-HNE AUC/FKBP12 AUC in the right panel.

The intracellular redox-state influences intermolecular and intramolecular disulfide bond formation as well as reduction and re-oxidation of mis-paired disulfide bonds. The intracellular redox-state is primarily regulated by the balance between oxidants (ROS) and the availability of antioxidants (free thiols including glutathione and cysteine). ROS, including hydrogen peroxide (H_2_O_2_) and superoxide anions (O_2_^−·^), are constantly generated intracellularly by enzymatic and non-enzymatic mechanisms. An imbalanced intracellular ROS accumulation disrupts the redox-homeostasis and may influence disulfide bond formation. Therefore, we determined relative changes in intracellular ROS levels in live cells from fed-batch and perfusion bioreactors using flow cytometry. Our results showed no significant differences in ROS levels between fed-batch and perfusion bioreactors during the exponential growth phase (MFI on day 9: 13,826 ± 444 vs. 12,518 ± 190) (Fig. 3A). However, ROS levels were dramatically lower during the stationary phase in perfusion bioreactors as compared to fed-batch bioreactors (MFI on day 15: 6986 ± 151 vs 18,337 ± 355). These data demonstrate that perfusion mitigates intracellular accumulation of ROS during the stationary phase.

Heavy metal ions such as copper, iron, zinc, lead and manganese also influence the intracellular redox environment. Moreover, these metal ions act as essential cofactors for a variety of intracellular enzymes and antioxidants that contribute to intracellular redox homeostasis. Excess intracellular accumulation of heavy metal ions due to an impaired redox metabolism can further exaggerate ROS production via a Fenton-type reaction, where H_2_O_2_ interacts with metal ions to form free hydroxyl (OH^−^) radicals^11^. Therefore, we next assessed relative changes in intracellular heavy metal ions to determine if perfusion had a differential effect on heavy metal ion uptake. Like ROS levels (Fig. 3A), no significant differences were found in intracellular heavy metal ion levels between fed-batch and perfusion processes during the exponential growth phase (Fig. 3B). However, intracellular metal ion levels were significantly higher in fed-batch bioreactors compared to perfusion bioreactors during the stationary phase (MFI on day 15: 584 ± 33 vs. 476 ± 6), which is concomitant to our findings in Fig. 3A. Together, these data demonstrate that perfusion caused lower intracellular accumulation of heavy metal ions during the stationary phase, contributing to lower intracellular ROS levels in perfusion bioreactors.

Intracellular ROS accumulation leads to lipid membrane peroxidation, oxidative modifications of proteins and DNA damage, ultimately leading to loss of structural function and cell death. 4-Hydroxynonenal (4-HNE), a cytotoxic reactive aldehyde is produced endogenously as a byproduct of the lipid membrane peroxidation and forms protein adducts with amino acid residues that disrupt protein structure and functions^12–14^. Studies have shown that 30% of 4-HNE adducted proteins are located in the mitochondria and a majority of them are the members of mitochondrial electron transport chain^15^. Therefore, we next analyzed cell samples from fed-batch and perfusion bioreactors for 4-HNE protein adduct formation as a marker of ROS-induced oxidative damage. We observed that 4-HNE adducts formation increased by ~ twofold from days 3 to 16 in the fed-batch bioreactor (Fig. 3C). In contrast, 4-HNE adducts formation in the perfusion process decreased by ~ threefold between days 3 and 14. Overall, 4-HNE adducts were considerably lower in the perfusion bioreactor when compared to a fed-batch bioreactor throughout the cell culture process.

Together, these data demonstrate that, relative to a perfusion process, a fed-batch process has elevated ROS levels triggering cellular oxidative damage. Elevated intracellular ROS accumulation in the fed-batch process disrupts intracellular redox-homeostasis and contributes to higher BisAb aggregate formation. In contrast, a perfusion process mitigates oxidative stress and protects CHO cells against moderate ROS-induced oxidative damage. Mitigation of intracellular ROS accumulation in the perfusion process contributes to improved intracellular redox-homeostasis and reduces BisAb aggregate formation.

### Perfusion process mitigates mitochondrial dysfunction-induced ROS production

**Figure 4:**
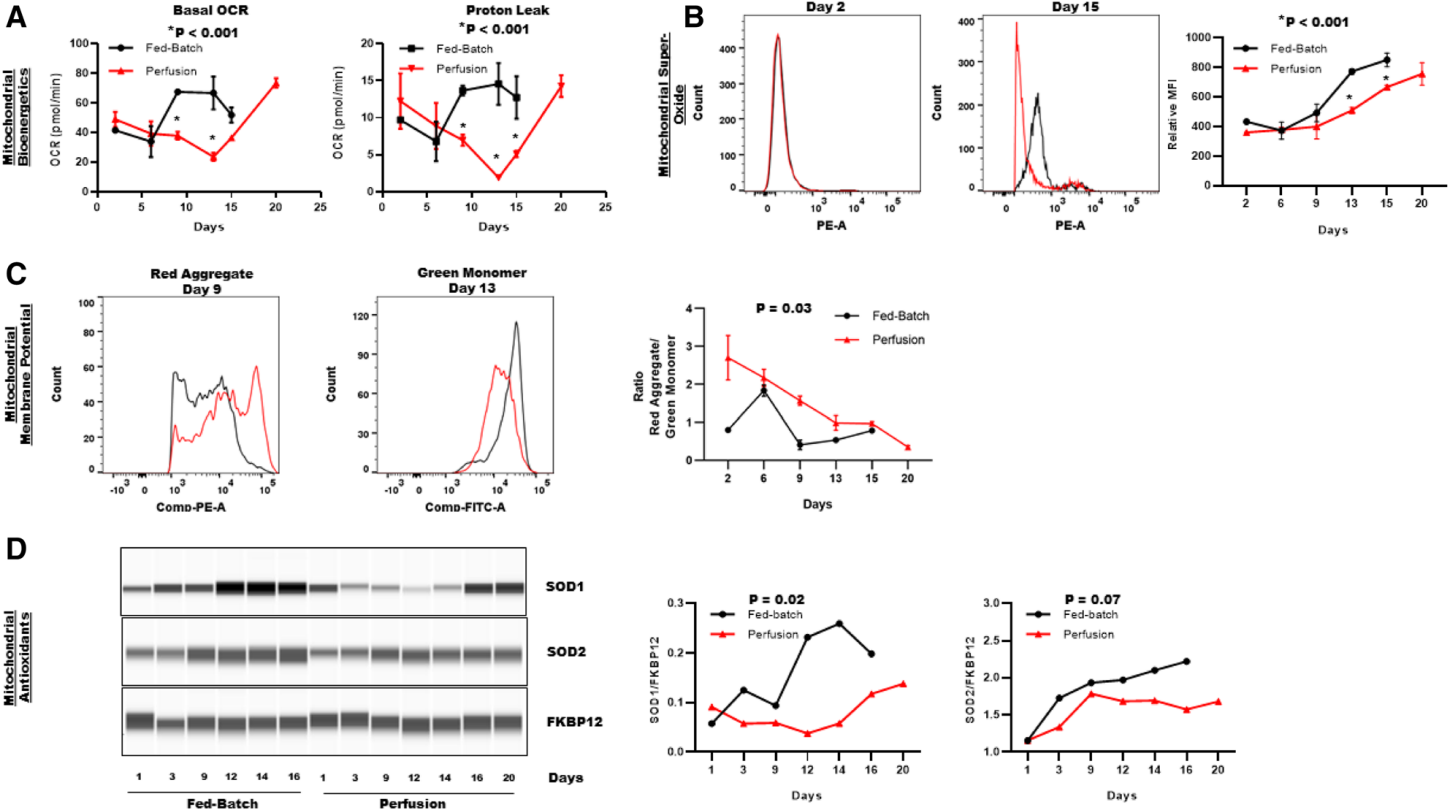
Analysis of mitochondrial function and superoxide production in fed-batch and perfusion processes. Cell samples were taken from each bioreactor on the indicated days to determine changes in (**A**) basal oxygen consumption (OCR) and proton leak (**B**) mitochondrial superoxide production (**C**) mitochondrial membrane potential and (**D**) superoxide dismutase expression (SOD1: ~ 16 kDa and SOD2: 22 kDa). Summarized seahorse and flowcytometry data are expressed as mean ± SD from duplicate bioreactors (**A**–**C**). (**D**) FKBP12 was used as loading control for the western blot analysis. Data is expressed as SOD AUC/FKBP12 AUC in the right panel.

Superoxide anions (O_2_^−·^) produced by the mitochondria are the precursor of most ROS^16^. Therefore, we determined the mitochondrial contribution to intracellular ROS production and evaluated mitochondrial bioenergetics influence on BisAb aggregate formation. In addition to ATP production by oxidative phosphorylation, the physiological function of the mitochondria also includes ROS generation and detoxification. Catalysis of O_2_^−·^ by antioxidant superoxide dismutases (SODs) in the mitochondria produces H_2_O_2_. However, oxidative stress can damage the mitochondrial electron transport chain (ETC) and the mitochondrial membrane which eventually leads to mitochondrial membrane depolarization^17^. Proton leak due to damaged mitochondrial membrane increases mitochondrial respiration (uncoupled respiration), further increasing superoxide production^16^. Therefore, to evaluate the effects of perfusion and fed-batch processes on mitochondrial bioenergetics and mitochondrial O_2_^−·^ production, we first examined the basal oxygen consumption rate (OCR) and uncoupled respiration due to proton leak using a Seahorse bioanalyzer (Fig. 4A). Both basal OCR and proton leak in fed-batch CHO cells were ~ twofold higher than perfusion CHO cells during the early stationary phase (basal OCR on day 9: 67.4 ± 1.7 vs. 37.8 ± 2.6 pmol/min and proton leak on day 9: 13.66 ± 0.6 vs. 6.9 ± 0.7 pmol/min). Interestingly, basal OCR started to decline between day 13 and day 15 (basal OCR: 51.8 ± 5 pmol/min) in fed-batch bioreactors without affecting proton leak. This data demonstrates a deteriorating cellular health due to damage to the inner mitochondrial membrane and/or ETC complex. In contrast, basal OCR levels in perfusion bioreactors began to increase from day 13 and remained elevated till day 20 (basal OCR: 73.4 ± 3 pmol/min). No significant differences in OCR or proton leak were observed between processes on day 1 and 5. These data demonstrate that the mid-process increase in basal OCR in fed-batch bioreactors is likely a combined effect of both higher specific productivity and 4-HNE-induced ETC dysfunction, therefore, increasing both mitochondrial ATP-linked respiration and uncoupled respiration respectively. On the other hand, mitigation of ETC dysfunction and lower specific productivity (Fig. 1C) enables perfusion bioreactor-cells to meet their ATP demand with lower OCR, without damaging the mitochondria.

Next, we compared mitochondrial superoxide production in live CHO cells from fed-batch and perfusion bioreactors. Super oxides are produced when oxygen inappropriately reacts with leaky electrons from the mitochondrial respiratory chain. It is well known that mitochondrial dysfunction underlies excessive mitochondrial superoxide production and increases cellular vulnerability to oxidative damage from ROS. Our analysis revealed that the fed-batch process caused significantly higher superoxide production in comparison to the perfusion process (Fig. 4B). Specifically, superoxide production during the stationary phase was more than 1.5-fold higher in fed-batch bioreactors compared to perfusion bioreactors (MFI on day 13: 772.5 ± 7 vs. 509.5 ± 14.5). Additionally, we also determined relative changes in mitochondrial membrane potential (Δψm) to gain further insight on how fed-batch and perfusion processes affect mitochondrial health (Fig. 4C). Throughout the cell culture process, perfusion bioreactor CHO cells had relatively higher mitochondrial membrane potential compared to fed-batch bioreactors. These data demonstrate that the perfusion process mitigates mitochondrial super oxide production and protects cells against mitochondrial oxidative damage and subsequent membrane depolarization.

Lastly, to further confirm our findings that the fed-batch process caused significantly higher superoxide production in comparison to the perfusion process (Fig. 4B), we investigated the effects of fed-batch and perfusion processes on intracellular antioxidant enzymes (SODs) that eliminate superoxide produced in the mitochondria (Fig. 4D). SOD1 is located in the cytoplasm and in the mitochondrial intermembrane space where superoxide anions are primarily generated due to leaky ETC whereas, SOD2 is located in the mitochondrial matrix^16^. Our data demonstrate that the protein expression of both SOD1 and SOD2 were higher in fed-batch bioreactors compared to perfusion bioreactors throughout the process, which is in agreement with our findings in Fig. 4B. Specifically, induction of superoxide production due to mitochondrial ETC dysfunction led to a dramatic upregulation of SOD1 expression to protect mitochondria from oxidative damage in fed-batch bioreactors. During the stationary phase of the culture process, fed-batch bioreactors showed more than fourfold increase in SOD1 expression compared to perfusion bioreactors (Fig. 4D). Although SOD2 expression was higher in fed-batch bioreactors, no significant differences in SOD2 expression were observed between fed-batch and perfusion bioreactors.

Taken together, these data demonstrate that the perfusion process enhances mitochondrial longevity by decreasing cellular ATP demand and mitigates ETC dysfunction-induced superoxide production. Mitigation of mitochondrial superoxide production decreases mitochondrial contribution to cumulative ROS production and decreases BisAb aggregation in perfusion bioreactors.

### Perfusion process improves glutathione regulation in CHO cells

**Figure 5:**
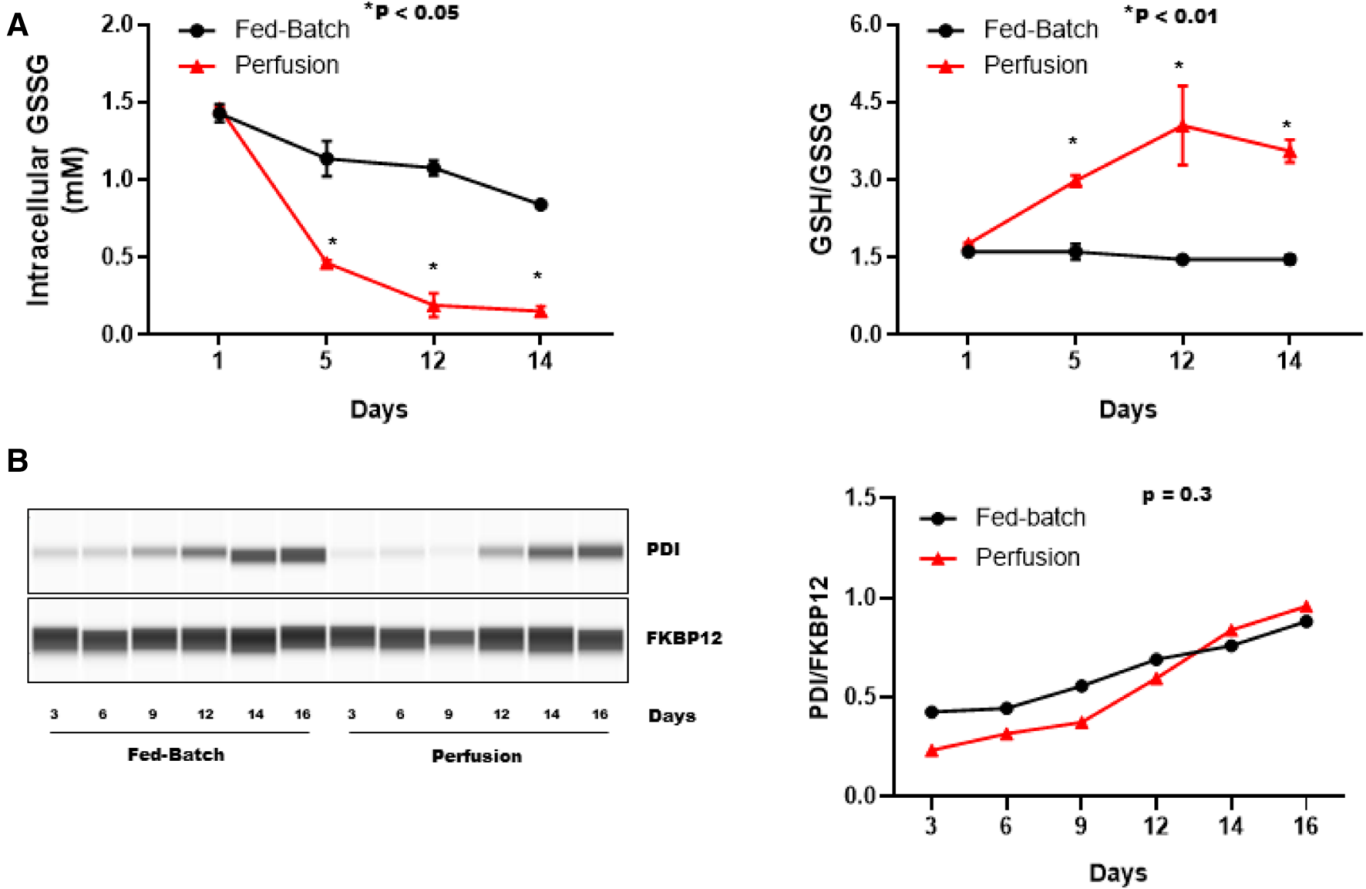
Analysis of the intracellular redox-state. (**A**) Cell samples were taken on the indicated days from each bioreactor and intracellular redox state was determined by measuring relative changes in intracellular glutathione concentration. Intracellular concentration of oxidized glutathione (GSSG) in fed-batch and perfusion bioreactors is shown in the left panel. Ratio of reduced glutathione (GSH) to GSSG is shown in the right panel. A higher ratio of GSH/GSSG is indicative of reduced intracellular redox-state. All data are expressed as mean ± SD. (**B**) Cell pellets were collected on the indicated days from a fed-batch and a perfusion bioreactor to determine changes in protein disulfide isomerase (PDI) (~ 57 kDa). Representative western blot image is shown in the left panel. Data is expressed as PDI AUC/FKBP12 AUC in the right panel.

The intracellular redox state influences disulfide bond formation during protein folding as well as mis-paired disulfide bond reduction and reformation in the ER. Cellular redox potential is largely regulated by intracellular glutathione levels. In addition to ROS detoxification in the cytosol, reduced glutathione transport to the ER is critical for the formation or reduction of disulfide bonds. In the ER, oxidized glutathione drives protein disulfide isomerase (PDI)-mediated disulfide bond formation^18^ while, reduced glutathione maintains PDI in a reduced state and catalyzes the reduction of mis-paired disulfide bonds^19^. Therefore, a reducing cytosolic environment is critical for reduced glutathione transport to the ER, maintaining ER-redox homeostasis, and ensuring reduction of mis-paired disulfide bonds that are formed due ER stress^20^. Disulfide bonds are formed when PDI oxidizes cysteine residues within the protein by accepting electrons from cysteine residues (Fig. 7). ER oxidoreductin 1 (ERO1) then re-oxidizes PDI by transferring electrons from PDI to molecular oxygen to form H_2_O_2_. Therefore, an overwhelming folding rate, due to high specific productivity, can trigger excessive H_2_O_2_ production in the ER, further exaggerating glutathione oxidation^21^ To determine how fed-batch and perfusion processes affect intracellular glutathione and disulfide bond formation, we assessed the ratio of reduced (GSH) to oxidized (GSSG) glutathione in CHO cells utilizing a luminescence-based assay (Fig. 5A). We observed that the relative intracellular concentration of GSSG was ~ fivefold higher in fed-batch bioreactors in comparison to perfusion bioreactors during the stationary phase (GSSG concentration on day 12: 1.07 ± 0.04 mM vs. 0.192 ± 0.07 mM). These data suggest an oxidizing intracellular environment in fed-batch bioreactors during the stationary phase. Moreover, a marked decrease in GSSG levels were observed in perfusion bioreactors, after the perfusion was initiated on day 2. Perfusion improved the GHS/GSSG ratio (Fig. 5A right panel) by ~ threefold when compared to fed-batch process. We did not observe any significant change in the GSH/GSSG ratio throughout the process in fed-batch bioreactors. Next, we assessed if fed-batch and perfusion processes had a differential effect on PDI expression due to different specific productivity and aggregates (Fig. 5B). We did not observe any significant differences in PDI expression between fed-batch and perfusion bioreactors. Together, these findings demonstrate that induction of the perfusion process improves intracellular glutathione ratio and maintains a favorable intracellular redox homeostasis.

### Perfusion process alleviates ER stress in CHO cells

**Figure 6:**
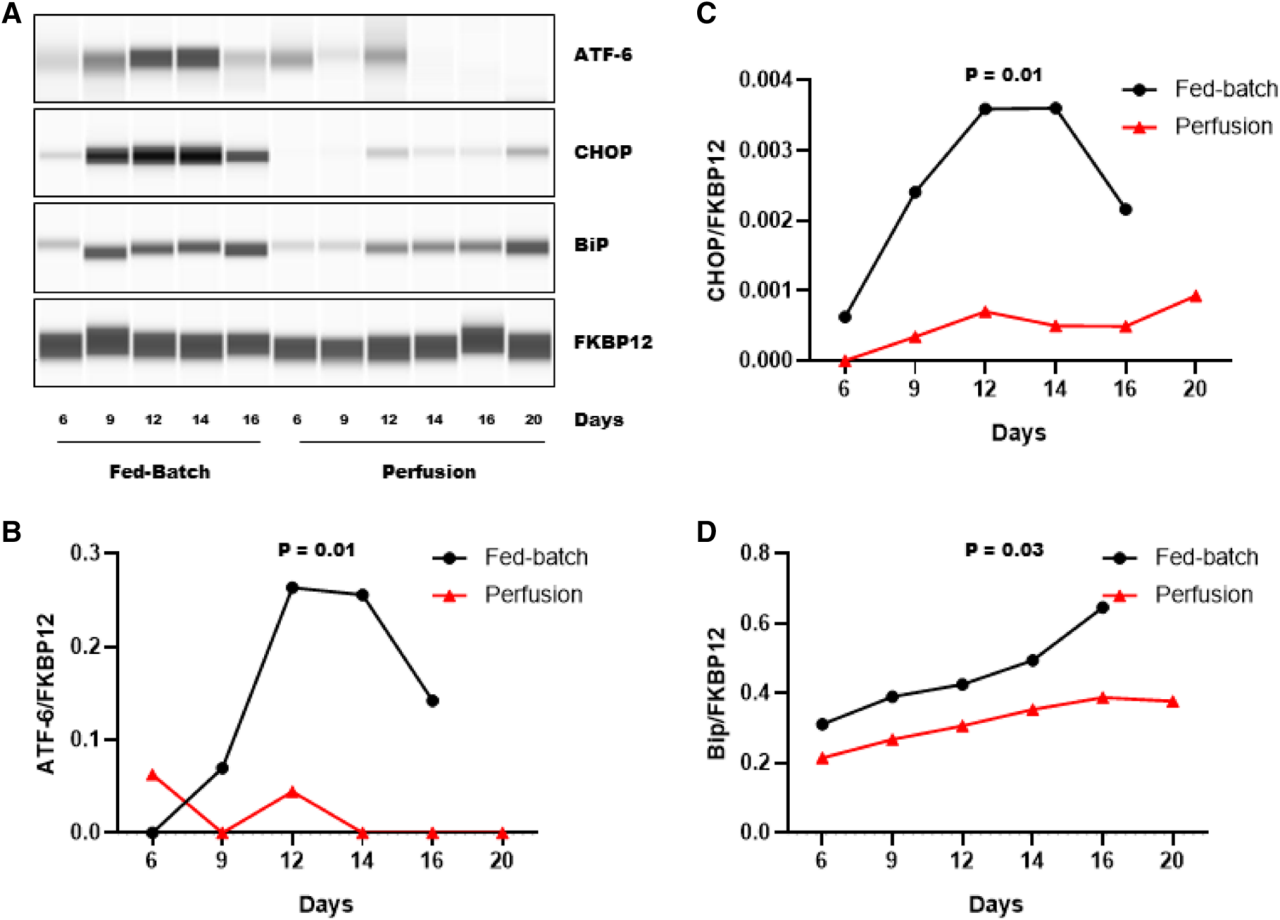
Analysis of ER stress in fed-batch and perfusion processes. Cell pellets were collected on the indicated days from a fed-batch and a perfusion bioreactor to determine protein expression of ATF-6, CHO and BiP using capillary western blot analysis. FKBP12 was used as a loading control. (**A**) Representative western blot images for ATF-6 (~ 100 kDa), CHOP (~ 27 kDa), BiP (~ 70 kDa) and FKBP12 (~ 12 kDa) are shown. (**B**–**D**) Densitometric analysis of western blot. Data are expressed as target protein AUC/loading control AUC.

Disulfide bonds are formed in the ER where a balanced redox-homeostasis is essential to catalyze disulfide bond formation and reduction in folded and misfolded proteins, respectively^21,22^. Oxidative stress or an overload in the protein folding rate due to high specific productivity disrupts ER homeostasis and is known as ER stress. Induction of ER stress activates a complex signaling network referred to as the Unfolded Protein Response (UPR), which helps withstand ER stress. During ER stress, ATF-6 bound BiP can detach itself from ATF-6 and bind exposed hydrophobic regions on the mis-folded protein. UPR responses also drive the upregulation of BiP, to promote refolding and alleviate ER stress (favorable UPR activation). If ER stress persist, UPR initiates apoptosis which is primarily mediated by CHOP. CHOP and ATF-6 expression are upregulated in response to sustained ER stress (unfavorable UPR activation)^21,23^. Therefore, to assess the effects of fed-batch and perfusion processes on ER stress, we determined changes in ATF-6, CHOP and BiP protein expression using western blot analysis (Fig. 6A–D). We found that the fed-batch process triggered an upregulation of ATF-6, CHOP and BiP protein expression, which were strikingly higher compared to the perfusion process. Specifically, during the stationary phase, the fed-batch process had an upregulation of ATF-6 by ~ threefold (Fig. 6B), CHOP by ~ threefold (Fig. 6C) and BiP by ~ 1.5-fold (Fig. 6D) compared to the perfusion process. We also noted that upregulation of both ATF-6 and CHOP in the fed-batch process starting from day 9 which is indicative of UPR-mediated cell apoptosis activation and is reflected in declining viability in the fed-batch process (Fig. 1B). Cumulatively, these findings demonstrate that the perfusion process leads to favorable UPR activation and mitigates UPR -mediated cell apoptosis. Perfusion-induced mitigation of ER stress improves ER homeostasis and mitigates BisAb aggregate formation.

## Discussion

**Figure 7:**
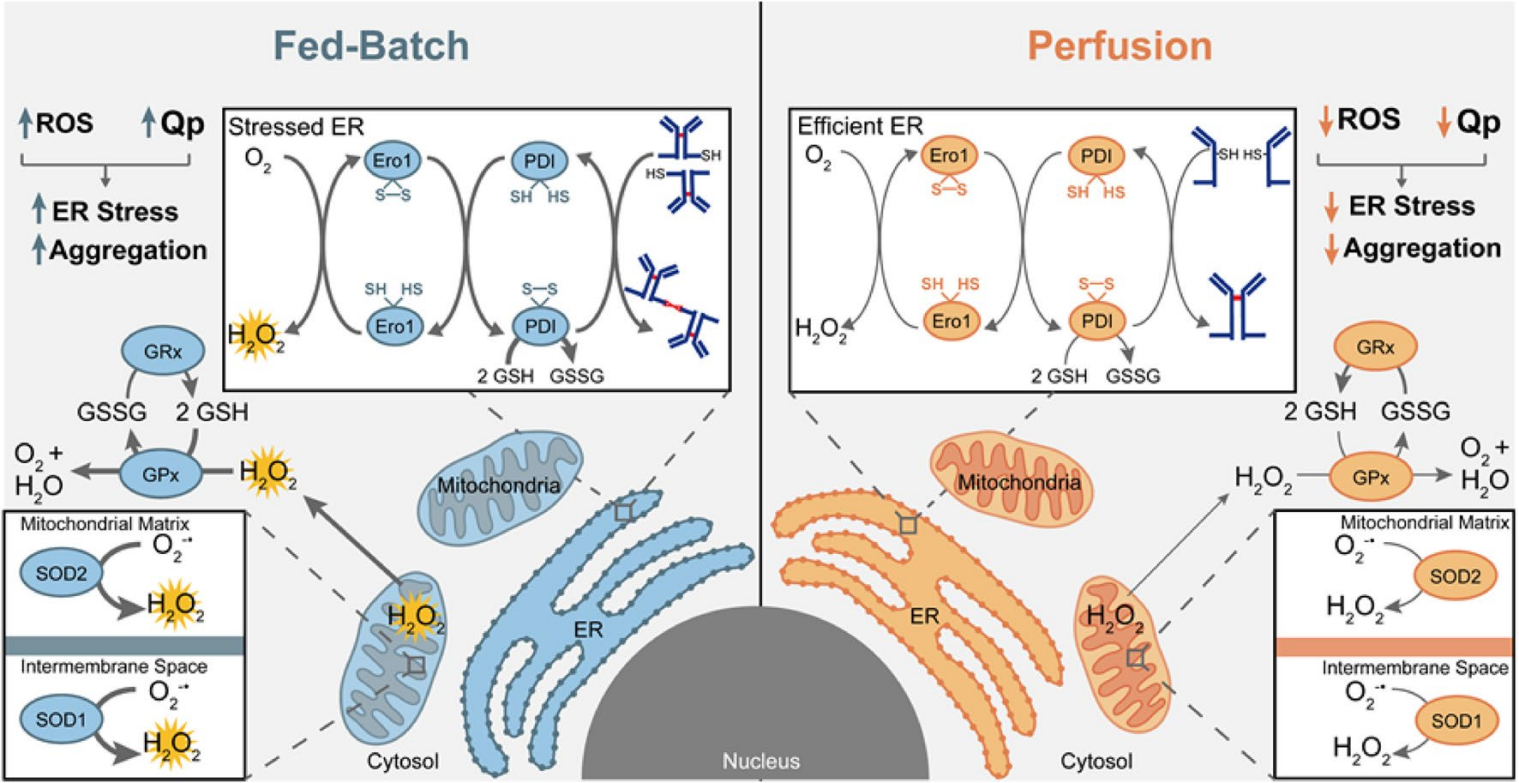
Schematic representation of the intracellular mechanisms underlying BisAb aggregation in a CHO cell process. Intracellular mechanistic differences between a fed-batch (Left) and a perfusion process (Right) are depicted. Left Panel: Reactive oxygen species (ROS) accumulation, caused by higher specific productivity (Qp) and oxidative damage, impairs intracellular glutathione regulation and disrupts ER homeostasis, leading to higher BisAb aggregation. Right Panel: Mitigation of intracellular ROS accumulation alleviate glutathione oxidation and improve reduced to oxidized glutathione ratio. Efficient intracellular glutathione regulation and lower Qp in a perfusion process protect CHO cells against ER stress and mitigates BisAb aggregation. This schematic illustration was made with Adobe Illustrator 23.0 software (https://www.adobe.com/products/illustrator.html).

At present we lack knowledge on the intracellular mechanisms and regulators that influence product aggregate formation. Therefore, in this study we utilized fed-batch and perfusion processes as tools to demonstrate that a synergistic coupling between cell specific productivity and intracellular redox-state tightly regulate intracellular product aggregate formation. We found that the perfusion process had a lower specific productivity and produced lower BisAb aggregate levels. In comparison, the fed-batch process had a higher specific productivity and produced higher BisAb aggregate levels. As mentioned earlier, BisAbs contain engineered disulfide bonds which make this type of antibodies more susceptible to aggregation due to disulfide bond mispairing^4^. Antibody disulfide bond formation in the ER is influenced by the intracellular redox state. Therefore, we compared both fed-batch and perfusion process at the intracellular-redox level to determine mechanisms leading to BisAb aggregate formation. It is well established that mitochondria are the primary source of intracellular ROS production^16^ and therefore, are a major regulator of the intracellular redox-state. We discovered the imperative role of efficient mitochondrial function in regulating the intracellular redox state, contributing to BisAb aggregation (Fig. 7). Mitochondrial ROS production is primarily regulated by the ETC and oxygen consumption^17^. Majority of consumed oxygen is utilized by the mitochondria for ATP production^24^. However, proton leak through the membrane due to damage of the mitochondrial membrane and ETC, further increase oxygen consumption (uncoupled respiration) and exaggerate ROS production. According to our analysis of mitochondrial function in relation to intracellular redox-state, we observed that the specific oxygen consumption needed to meet cellular ATP demand was significantly less due to lower specific productivity in perfusion bioreactors (Fig. 7). As a result, lower levels of superoxide were formed in the mitochondria which indicates that the perfusion process decreases mitochondrial load and mitigates ETC dysfunction. Therefore, perfusion-induced mitigation of mitochondrial superoxide production decreases mitochondrial contribution to overall ROS generation. Thus, the perfusion process protects CHO cells against oxidative damage from lipid peroxidation and subsequent mitochondrial damage and depolarization. The perfusion process also limits cellular uptake of heavy metal ions which are also known to further exaggerate oxidative stress via a Fenton-type reaction^11^.

The glutathione redox couple GSH and GSSG play a critical role in maintaining intracellular redox-homeostasis and disulfide bond formation in the ER. Moreover, GSH transport to the ER is essential to catalyze the reduction of the non-native disulfide bonds that are formed due to ER-stress. Our data conclusively demonstrate that the perfusion-induced mitigation of ROS production and accumulation enables CHO cells in perfusion bioreactors to maintain a better ratio of GSH to GSSG. A favorable intracellular redox environment in the perfusion process facilitates proper protein folding and the reduction of aggregated proteins in the ER (Fig. 7). Comparatively, higher glutathione oxidation in fed-batch bioreactors is likely a combined effect of higher ROS production from both mitochondria and ER due to higher specific productivity and protein folding load respectively. Moreover, under ER stress, the activation of UPR signaling cascade upregulates the catalytic activities of BiP to further promote oxidative folding and increase mis-paired disulfide bond formation, ultimately leading to BisAb aggregate formation in the fed-batch process^25^. Although, our current study is limited to PDI expression instead of PDI activity, no significant differences in PDI expression were observed between processes. Together, these data demonstrate that oxidative stress-induced exaggerated glutathione oxidation mitigates mis-paired disulfide bond reduction causing BisAb aggregation intracellularly. Moreover, ER stress-induced UPR activation upregulates protein folding and mis-paired disulfide bond formation, further increasing BisAb aggregate formation in the fed-batch process.

In conclusion, our findings elucidate that the perfusion bioreactors maintain a more favorable intracellular environment than the fed-batch bioreactors and provide us novel insights into fundamental intracellular mechanisms contributing to BisAb aggregate formation. In this study we conclusively demonstrated how changes in culture processes and the intracellular environment can dramatically affect mitochondrial health, redox homeostasis, and ER function, synergistically regulating product aggregate formation. To our knowledge, this is the first study to demonstrate the regulatory effects of mitochondrial-ER activity on product quality, specifically for BisAb production. Our study clearly demonstrates the importance of remote-monitoring intracellular redox state along with extracellular parameters during process development. Since, efficient mitochondrial function and glutathione metabolism is critical for a robust cell culture process, intracellular redox state should be monitored to develop next-generation platform processes that will reduce product aggregate formation. Knowledge gained from this study can also be implemented for cell line engineering and clone selection processes based on the intracellular redox-state. This can further improve overall cell productivity and reduce product aggregates. Lastly, our observations related to the interplay between intracellular redox state and product aggregate formation is likely applicable for the process development of all therapeutic proteins containing disulfide bonds including antibody–drug conjugates, mAb-peptide conjugates and fusion proteins.

## Methods

### CHO cell culture process and antibody production

For all the experiments related to this work, the same clonal CHO cell line expressing a BisAb was used. These cells were cultured in duplicate 3 l glass stirred tank bioreactors (2 biological replicate/group) either in a fed-batch^26^ or perfusion process^27^ as previously described, with an initial working volume of 1.5 l. Cells were cultured under conditions representative of large scale manufacturing processes and are outlined in Table 1. Temperature, agitation, dissolved oxygen and pH were monitored and controlled using DASGIP controller software (Eppendorf, Germany). Fresh cell samples were collected every day for subsequent process analysis and biochemical assays. Offline pH, gases (pCO_2_ and O_2_), lactate and glucose levels were analyzed using RAPIDPOINT 500 Blood Gas Analyzer (Siemens, PA). Viable cell density and cell viability were determined using Vi-CELL XR (Beckman Coulter, CA). Osmolality was measured using an osmometer (Advanced Instruments Inc., MA). Titer levels for each bioreactor were quantified on 4.6 × 50 mm Protein A columns (ThermoFisher Scientific, MA) using HPLC (Agilent Technologies, CA).

### Quantification and detection of aggregate levels

Cell free samples collected from each bioreactor were evaluated for total aggregation by ultra-performance size exclusion chromatography with an ACQUITY UPLC Protein BEH SEC column, 200 Å, 1.7 µm, 4.6 mm × 300 mm (Waters Corp., MA) and an Agilent 1200 HPLC system (Agilent Technologies, CA). Intracellular aggregate levels were determined using western blot analysis under reducing and non-reducing conditions as described below.

### Detection of intracellular ROS, mitochondrial superoxide, mitochondrial membrane potential and intracellular heavy metal ions

Relative intracellular changes in ROS, mitochondrial superoxide, mitochondrial membrane potential (Ψm) and heavy metal ions were detected using flow cytometry according to manufacturer’s instruction and previously performed^26,28^. Briefly, 1 × 10^6^ CHO cells were harvested from duplicate bioreactors by centrifugation at 300×*g* for 5 min. Cells were washed with either HBSS or 1 × Mito-ID assay buffer. Cells were then stained with either CM-H_2_DCFDA (ThermoFisher Scientific, MA), or MitoSox Red (ThermoFisher Scientific, MA) or Phen Green FL (ThermoFisher Scientific, MA) or Mito-ID detection reagent (Enzo Life Sciences, NY) for 15 min at room temperature in the dark room. Stained cells were analyzed using a LSRII cytometer (BD Biosciences, CA). Flowcytometry data were analyzed using FlowJo software 10.6 (Tree Star, Inc., OR). For mitochondrial membrane potential analysis bivariate plots of green versus red fluorescence as well as univariate histogram plots of red fluorescence were used. The red fluorescence emission indicates energized mitochondria with high membrane potential whereas, the green fluorescence emission associated with depolarized mitochondria.

### Capillary western blot analysis

10^6^ cells were lysed in RIPA protein extraction reagent containing Halt Protease and Phosphatase Inhibitor Cocktail (ThermoFisher Scientific, MA) and protein concentrations were determined using Pierce BCA assay kit (ThermoFisher Scientific, MA) following manufacturer’s instruction and previously performed^29^. Western blot analysis was performed using a WES system and anti-rabbit detection modules (Protein Simple, CA) with either 12–220 kDa or 66–440 kDa (25 well cartridge) separation module as appropriate and previously performed^30^. All samples were diluted to a final loading concentration of 1 μg/µl and equal amount of total protein (5 μg) were loaded for each sample. For non-reducing western blot analysis protein samples were prepared without DTT. Samples were run using following instrument settings: separation at 375 V for 25 min, blocking for 30 min, primary and secondary antibody incubation 30 min and chemiluminescence detection for 15 min (exposures: 1–2–4–8–16–32–64–128–512 s). Peroxidase AffiniPure Goat Anti-Human IgG, Fcγ fragment specific antibody was purchased from Jackson ImmunoResearch (PA). Rabbit primary antibodies against CHOP, Bip, ATF-6, PDI and SOD2 were purchased from Cell Signaling Technologies (MA). Rabbit primary antibodies against SOD1 and 4-HNE were obtained from Abcam (MA) and Alpha Diagnostic Inc. (TX) respectively. For all western blot studies day-matched samples from a fed-batch and perfusion bioreactor were run in the same cartridge. FKBP12 (ThermoFisher Scientific, MA) was used as a loading control when appropriate to normalize peak intensities between capillaries^31^.

### Glutathione assay

Relative changes in intracellular reduced glutathione (GSH) and oxidized glutathione (GSSG) levels were determined with GSH/GSSG-Glo Assay kit (Promega, WI). Briefly, live cells collected from duplicate bioreactors were rinsed and resuspended in HBSS buffer, then seeded at 10,000 cells/well in a 96-well luminometer-compatible plate. 25 µl of either total glutathione lysis reagent or oxidized glutathione lysis reagent was added to the wells containing cells and incubated at room temperature on a plate shaker for 5 min. Then 50 µl of freshly prepared luciferin generation reagent was added to all the wells followed by 30 min incubation at room temperature. 100 µl of luciferin detection reagent was then added to each well. Luminescence was measured after 15 min of incubation using a LUMIstar Omega Microplate Luminometer (BMG Labtech). No cell and HBSS buffer only wells were used for background luminescence detection. Relative Luminescence Unit (RLU) for GSH levels for each bioreactor samples were determined by subtracting RLU of GSSG from RLU of total glutathione.

### Seahorse mitochondrial stress analysis

Mitochondrial function was evaluated using a Seahorse XF96e flux analyzer (Agilent Technologies, CA) as previously performed^32^. All reagents for flux analysis were purchased from Agilent Technologies unless otherwise noted. Oxygen consumption rates (OCR) measurements were collected using the mitochondrial stress test kit in XF DMEM media containing 10 mM glucose, 1 mM pyruvate and 2 mM l-glutamine and upon addition of 1uM oligomycin, 1uM FCCP and 0.5uM rotenone/antimycin A. Cells were used immediately following collection from duplicate bioreactors. Briefly, cells were rinsed and resuspended in XF media, then seeded at 30,000 cells per well onto the wells of coated 96-well XF Cell Culture Microplates. Assay plates were coated with 25 µl of 22.4 µg/ml Cell-Tak (VWR, PA) solution for 20 min and washed twice with sterile water. Coated plates were used within 1 week, stored at 4 °C and allowed to warm to room temperature prior to cell seeding. Once seeded, plates were centrifuged at 200×*g* for 1 min with zero braking to facilitate attachment of cells and then topped off to a final volume of 180 µl with XF media. Measurements were collected on the Seahorse XF96e flux analyzer and analysis was provided by the accompanying Wave software (Version 2.6.0) and built-in Seahorse XF Cell Mito Stress Test Report Generator.

### Statistical analysis

Sample size chosen for this study were based on our previously published experiences^26^. All data are expressed as mean ± SD. Statistical significance was assumed for p < 0.05. Statistical significance for all cell culture process data and western blot analysis was performed using unpaired t-test with Welch’s correction as parametric test. All other assay data were analyzed with multiple t-test with Bonferroni-Dunn method for multiple comparison correction. All statistical analysis was performed using GraphPad Prism 8.0 (GraphPad Software, CA). Schematic illustration was made with Adobe Illustrator 23.0. software (Adobe, CA).

## Supplementary information

Supplementary information.

## Data Availability

All data relevant to this study are provided within the manuscript.

## References

[1] Labrijn AF, Janmaat ML, Reichert JM, Parren P (2019). Bispecific antibodies: A mechanistic review of the pipeline. Nat. Rev. Drug Discov..

[2] Spiess C, Zhai Q, Carter PJ (2015). Alternative molecular formats and therapeutic applications for bispecific antibodies. Mol. Immunol..

[3] Brinkmann U, Kontermann RE (2017). The making of bispecific antibodies. MAbs.

[4] Cao M (2018). Characterization and analysis of scFv-IgG bispecific antibody size variants. MAbs.

[5] Swope N (2020). Impact of enzymatic reduction on bivalent bispecific antibody fragmentation and loss of product purity upon reoxidation. Biotechnol. Bioeng..

[6] Qian Y, Jing Y, Li ZJ (2010). Glucocorticoid receptor-mediated reduction of IgG-fusion protein aggregation in Chinese hamster ovary cells. Biotechnol. Prog..

[7] Dimasi N, Fleming R, Wu H, Gao C (2019). Molecular engineering strategies and methods for the expression and purification of IgG1-based bispecific bivalent antibodies. Methods.

[8] Gomez N (2019). Perfusion CHO cell culture applied to lower aggregation and increase volumetric productivity for a bispecific recombinant protein. J. Biotechnol..

[9] Zhu MM (2005). Effects of elevated pCO2 and osmolality on growth of CHO cells and production of antibody-fusion protein B1: a case study. Biotechnol. Prog..

[10] Cromwell ME, Hilario E, Jacobson F (2006). Protein aggregation and bioprocessing. AAPS J..

[11] Leonard SS, Harris GK, Shi X (2004). Metal-induced oxidative stress and signal transduction. Free Radic. Biol. Med..

[12] Esterbauer H, Schaur RJ, Zollner H (1991). Chemistry and biochemistry of 4-hydroxynonenal, malonaldehyde and related aldehydes. Free Radic. Biol. Med..

[13] DelloStritto DJ (2016). 4-Hydroxynonenal dependent alteration of TRPV1-mediated coronary microvascular signaling. Free Radic. Biol. Med..

[14] Sinharoy P, McAllister SL, Vasu M, Gross ER (2019). Environmental aldehyde sources and the health implications of exposure. Adv. Exp. Med. Biol..

[15] Poli G, Schaur RJ, Siems WG, Leonarduzzi G (2008). 4-Hydroxynonenal: A membrane lipid oxidation product of medicinal interest. Med. Res. Rev..

[16] Sena LA, Chandel NS (2012). Physiological roles of mitochondrial reactive oxygen species. Mol. Cell.

[17] Turrens JF, Freeman BA, Levitt JG, Crapo JD (1982). The effect of hyperoxia on superoxide production by lung submitochondrial particles. Arch. Biochem. Biophys..

[18] Merksamer PI, Trusina A, Papa FR (2008). Real-time redox measurements during endoplasmic reticulum stress reveal interlinked protein folding functions. Cell.

[19] Jessop CE, Bulleid NJ (2004). Glutathione directly reduces an oxidoreductase in the endoplasmic reticulum of mammalian cells. J. Biol. Chem..

[20] Molteni SN (2004). Glutathione limits Ero1-dependent oxidation in the endoplasmic reticulum. J. Biol. Chem..

[21] Zhang K, Kaufman RJ (2008). From endoplasmic-reticulum stress to the inflammatory response. Nature.

[22] Chaudhuri P (2016). Membrane translocation of TRPC6 channels and endothelial migration are regulated by calmodulin and PI3 kinase activation. Proc. Natl. Acad. Sci. U.S.A..

[23] Pierre N (2014). Activation of ER stress by hydrogen peroxide in C2C12 myotubes. Biochem. Biophys. Res. Commun..

[24] Muller B (2019). Application of extracellular flux analysis for determining mitochondrial function in mammalian oocytes and early embryos. Sci. Rep..

[25] Travers KJ (2000). Functional and genomic analyses reveal an essential coordination between the unfolded protein response and ER-associated degradation. Cell.

[26] Handlogten MW (2018). Intracellular response to process optimization and impact on productivity and product aggregates for a high-titer CHO cell process. Biotechnol. Bioeng..

[27] Arnold L, Lee K, Rucker-Pezzini J, Lee JH (2019). Implementation of fully integrated continuous antibody processing: Effects on productivity and COGm. Biotechnol. J..

[28] Cossarizza A (2009). Simultaneous analysis of reactive oxygen species and reduced glutathione content in living cells by polychromatic flow cytometry. Nat. Protoc..

[29] Sinharoy P (2017). TRPA1 and TRPV1 contribute to propofol-mediated antagonism of U46619-induced constriction in murine coronary arteries. PLoS ONE.

[30] Nguyen U, Squaglia N, Boge A, Fung PA (2011). The simple Western^TM^: A gel-free, blot-free, hands-free Western blotting reinvention. Nat. Methods.

[31] Brown AJ, Gibson S, Hatton D, James DC (2018). Transcriptome-based identification of the optimal reference CHO genes for normalisation of qPCR data. Biotechnol. J..

[32] Wu M (2007). Multiparameter metabolic analysis reveals a close link between attenuated mitochondrial bioenergetic function and enhanced glycolysis dependency in human tumor cells. Am. J. Physiol.-Cell Physiol..

