# Supplementary material for: Perfusion reduces bispecific antibody aggregation via mitigating mitochondrial dysfunction-induced glutathione oxidation and ER stress in CHO cells
Source: Sci Rep. 2020 Oct 6;10:16620. doi: 10.1038/s41598-020-73573-4 (PMC7538420; doi:10.1038/s41598-020-73573-4)
Supplement: Supplementary file 1 — Supplementary information. [file 41598_2020_73573_MOESM1_ESM.pdf]

Perfusion Reduces Bispecific Antibody Aggregation via Mitigating Mitochondrial Dysfunction-Induced Glutathione Oxidation and ER Stress in CHO Cells

Pritam Sinharoy[1], Aaron H. Aziz[1], Natalia I. Majewska[1 2], Sanjeev Ahuja[1], Michael W. Handlogten[1]*.

[1]Cell Culture and Fermentation Sciences, AstraZeneca, MD, USA

[2]Department of Chemical and Biomolecular Engineering, Whiting School of Engineering, Johns Hopkins University, MD, USA

Supplementary Figure 1

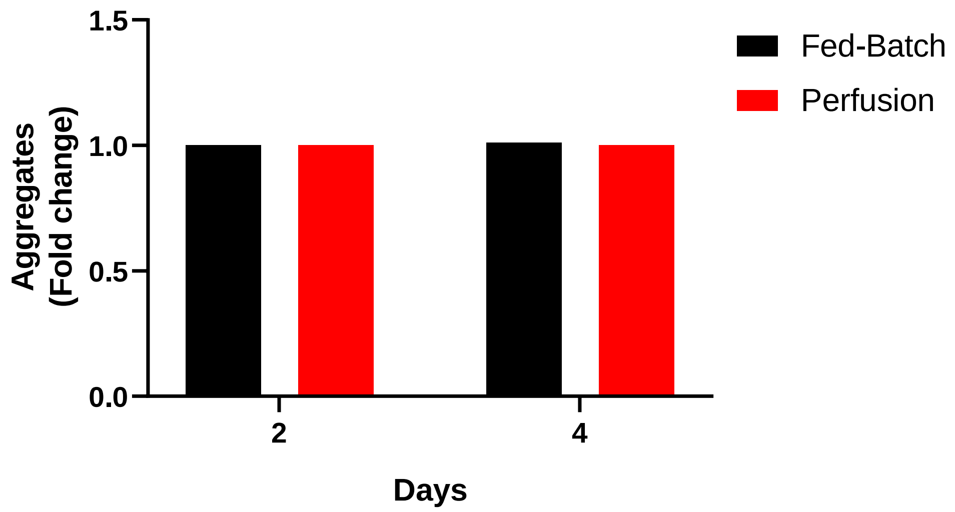

Supplementary Figure 1: Cell free samples collected from a fed-batch and a perfusion bioreactor on day 16 were incubated at 35.5°C for 4 days. The effect of the culture medium on extracellular aggregate formation was determined by comparing changes in aggregate levels between day 2 and day 4 samples. Data are expressed as fold-change in comparison to day 2.

Supplementary Figure 2

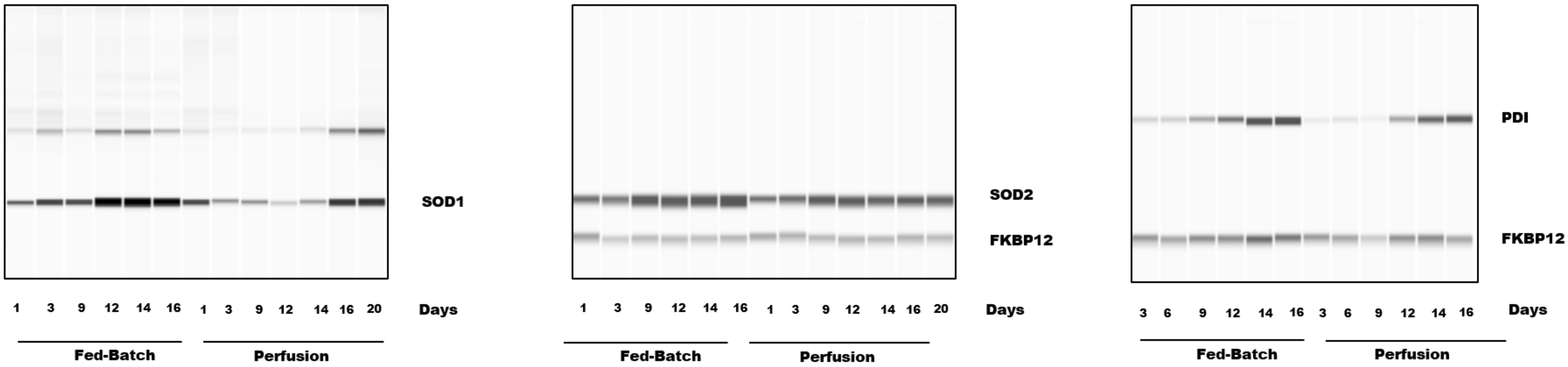

Supplementary Figure 2: Representative full images of the capillary western blot analysis for SOD1, SOD2 and PDI protein expression in a fed-batch and a perfusion bioreactor.

Supplementary Figure 3

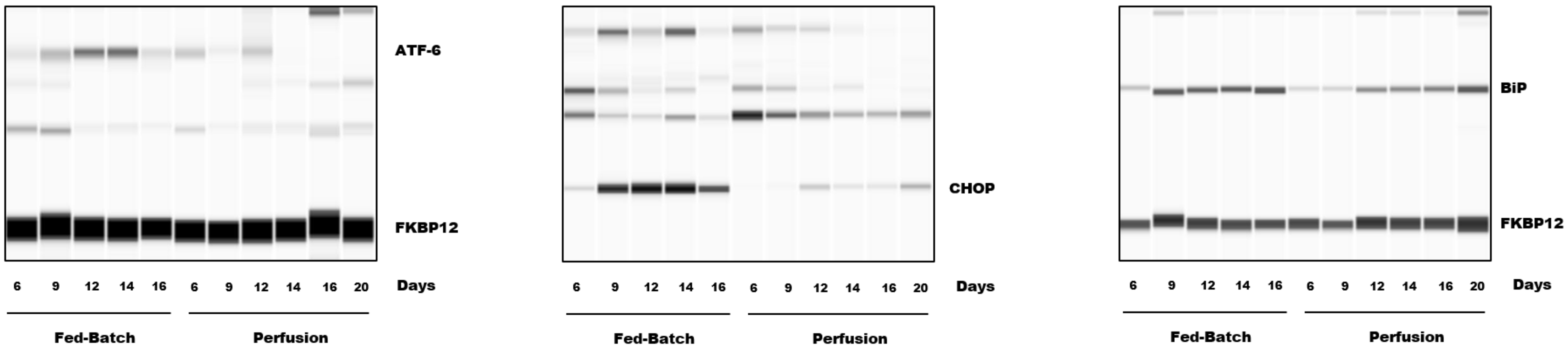

Supplementary Figure 3: Representative full images of the capillary western blot analysis for ATF-6, CHOP and BiP protein expression in a fed-batch and a perfusion bioreactor.
